# Editorial Notes

**Published:** 1893-05

**Authors:** 


### Editorial Notes.

DEATH FROM NITROUS OYIDE GAS.—A sudden death in a dentist's chair is reported from Buffalo, due to inhalation of nitrous oxide gas.

AMERICAN GYNECOLOGICAL SOCIETY.—The eighteenth annual meeting of this society will be held in the College of Physicians, corner 13th and Locust streets, Philadelphia, on May 16, 17 and 18, 1893.

THE ASSOCIATION OF AMERICAN MEDICAL COLLEGES.—The fourth annual session of this association will be held at the Pfister Hotel, Milwaukee, Wis., on Wednesday, June 7, at 3 P. M. Amendments to the by-laws will be suggested, granting associate membership of one delegate from each recognized school of Post.graduate Instructioa in the United States, and from each State Board of Medical Examiners in the United States. It is also proposed to divide membership in the association into three classes, of active, associate, ann honorary.

THE COLORADO STATE MEDICAL SOCIETY.—The twenty-third annual meeting of this society is to be held in Denver, June 20, 21 and 22, 1893.

THE ILLINOIS STATE MEDICAL SOCIETY.—The forty-third annual meeting of this society will be held in Chicago, in the Methodist Church Block, corner of Washington and Clark Streets, on May 16, 17 and 18, 1893.

MEDICAL SOCIETY OF THE STATE OF PENNSYLVANIA.—The forty.third annual meeting will be held in Williamsport, on Tuesday, Wednesday, Thursday and Friday, May 16, 17, 18, and 19, 1893.

### BOOKS AND PAMPHLETS RECEIVED.

Extensive Railroad Injury; Amputation-Fistula in Ano-Peritonitis, by Thomas H. Manley, M. D., New York. (Reprint.)

Methods of Precision in the Investigation of disorders of Digestion, by J. H. Kellogg, M. D., Battle Creek, Mich.

Hypnotism as a Therapeutic Agent, by W. L. Howard, M. D., Baltimore.

The Sympathetic Nerve and Abdominal Brain in Gynecology; its Reflexes and its Rhythm, by F. B. Robinson, M. D., Chicago. (Reprint.)

Eye Troubles which Constitute a Frequent Source of Headache, Vertigo and Nausea, and other Nervous Disorders, by F. C. Hotz, M. D., Chicago. (Reprint.)

Ripening of Immature Cataracts by Direct Trituration, by B. Bettman, M. D., Chicago. (Reprint.)

Fixation after Excision of the Knee, by H. A. Wilson, M. D., Philadelphia.

"Oils and Fats" in Surgical Dressings, by C. M. Hobby, M. D., Iowa City. (Reprint.)

A Materialistic View of Sexual Impotence, by Bransford Lewis, M. D., St. Louis. (Reprint.)

Practical Experiments in the Treatment of Cholera in St. Petersburg, Russia and Hamburg, Germany, in the Epidemic of 1892, by Elmer Lee, M. D., Chicago. (Reprint.)

Surgical Therapy of Rectal Cancer, by T. H. Manley ,M. D., New York. (Reprint.)

The Value of Javal's Ophthalmometer for the Correction of Astigmatism where Marked Amblyopia is Present, by A. B. Beynard, M. D., New York. (Reprint.)

Brain Surgery: Injury Received Five Years Ago, Followed Three Years Later by Convulsions and Paralysis—Blood Clot Found under the Dura mater and Removed—Patient Improving, by F. C. Schaefer, M. D., Chicago, Ill, (Reprint.)

